# Injections of Camphorated Oil in Surgical Affections

**Published:** 1911-12

**Authors:** 


Injections of Camphorated Oil in Surgical Affections.—P. Baudet (*La Province Medicale*).

Baudet strongly recommends the oil injections in all surgical infections, the pneumonias and enteritis in children. The oil is a powerful cardiac stimulant and also possesses an antitoxic action.

In general, he injects 1c.c. of camphorated oil 10-100, two or three times a day. In grave infections the dosage may be increased to 50c.c. injected morning and evening, i.e., 100c.c. in 24 hours or 10 grams of pure camphor.

A Roux syringe is used and the oil is prepared according to the formula given by M. Valdiguier, Chief Pharmacist, Hotel Dieu.

1,000 c.c. olive oil are mixed with 300 c.c. 95% alcohol to remove the free acids, repeatedly shaken, allowed to stand 24 hours and decanted. This operation is repeated three times. The final product is carefully heated on a sand bath to remove all traces of alcohol.

100 grams of camphor are dissolved in 900 grams of the washed oil, filtered, placed in 10 and 20 c.c. ampules and sterilized in the autoclave. Each ampule contains ½ grams of pure camphor.

Hirschel of Heidelberg, in desperate cases of peritonitis from perforation, pours 100-300 c.c. of the oil into the peritoneal cavity before closing the abdominal wound. His object being to lubricate the abdominal contents, obstruct the lymphatics and delay absorption. He claims two actions for the drug, first bacteriocidal and second, mechanical in closing the lymphatic orifices; comparing its action to that of balsam of peru in infected wounds.

W.W.Q.

---

LEUCO-PROPHYLAXIS IN ITS APPLICATION TO ABDOMINAL SURGERY—O. L. Botero—*La Semana Medica,* Buenos Ayres, August, 1911.

Bottaro uses horse serum, and nucleinic acid 2% for this purpose. The serum is administered subcutaneously in doses of 10 c.c. or applied locally to the peritoneal cavity. The nucleinic acid is used in a similar way.

He has injected 30 c.c. of a 0.50% solution of the acid into the peritoneal cavity per vaginum and cul-de-sac of Douglas. The injections are made 18-24 hours before operation and are followed by no appreciable disturbance.

Acording to his experiments, following the injection of these substances there is a diminution in the number of leucocytes in the general circulation and an enormous increase in the number present in the peritoneal exudate. W.W.Q.

---

PETERKIN in an article entitled, "Urologic Facts," *International Journal of Surgery,* September, 1911, emphasizes the fact that the urethral walls are collapsed when at rest but when distended the canal has a capacity of 25 c.c. He questions the value of a 2 drachm syringe in medicating the entire surface of the canal and advocates the use of a douche bag and a glass nozzle.

He also insists that a full bladder is essential when palpating the seminal vesicles. The mechanics of cystocele and its relation to retroversion of the uterus and laceration of the perinaeum are also discussed.

The October number of *The Medical Council* contains an article by Bogart entitled, "One Venereal Menace." He mentions a venereal specialist in a university town who has treated one third of all the students in a single year and his records show that 90 per cent. of the graduates have been under his care in the past.

Another physician who practiced for 30 years in the vicinity of a co-educational school informs him that venereal diseases are rampant among the students of both sexes.

What stronger argument for instruction in sex hygiene, can one find than this.

